# The role of irrigation in changing wheat yields and heat sensitivity in India

**DOI:** 10.1038/s41467-019-12183-9

**Published:** 2019-09-12

**Authors:** Esha Zaveri, David B. Lobell

**Affiliations:** 1 Department of Earth System Science and Center on Food Security and the Environment, Stanford University, Stanford, CA 94305 USA; 2 Water Global Practice, The World Bank, Washington DC, 20433 USA

**Keywords:** Climate-change impacts, Environmental economics, Environmental impact, Sustainability

## Abstract

Irrigation has been pivotal in wheat’s rise as a major crop in India and is likely to be increasingly important as an adaptation response to climate change. Here we use historical data across 40 years to quantify the contribution of irrigation to wheat yield increases and the extent to which irrigation reduces sensitivity to heat. We estimate that national yields in the 2000s are 13% higher than they would have been without irrigation trends since 1970. Moreover, irrigated wheat exhibits roughly one-quarter of the heat sensitivity estimated for fully rainfed conditions. However, yield gains from irrigation expansion have slowed in recent years and negative impacts of warming have continued to accrue despite lower heat sensitivity from the widespread expansion of irrigation. We conclude that as constraints on expanding irrigation become more binding, furthering yield gains in the face of additional warming is likely to present an increasingly difficult challenge.

## Abstract (web summary)

Irrigation buffers crop yields from extreme weather, but comes at environmental costs. Here the authors show that in India irrigation has improved wheat yield and reduced its sensitivity to heat, yet further increases are unlikely to offset the impact of warming.

## Introduction

Wheat is one of the two main crops grown in India and ever since the Green Revolution it has played a critical role for both national and global food security. Globally, India is the second largest producer of wheat, exporting 0.2 million tons annually and contributing 13% of the wheat supply^1,2^. A fall in Indian wheat yield can, thus, directly affect global cereal prices. India’s agriculture sector is also the primary food supplier of India’s 1.3 billion people that make up 17.5% of the world’s population. Notably, wheat occupies the largest share of dry-season cultivated area, providing one-fifth of household calories and half of all calories obtained from cereals^3^. The impact of weather variability and climate change on wheat yields, therefore, remain central to food security concerns and directly affect the livelihood of small-scale farmers who control the majority of the landholdings in India, and produce 41% of India’s food grains^4^

Extreme heat remains especially detrimental to wheat production^5–7^. Prior work has shown that temperature increases due to climate change can reduce global wheat yields by up to 30 percent by mid-century^8,9^. This is of concern in India as average temperatures have been increasing and are projected to increase even further in the future^10^. Wheat yields have already fallen by up to 5 percent due to current warming^5,11^ and further reductions are likely with predicted future changes in temperature. In response, farmers can adjust their practices to adapt to changes in weather. One such strategy that Indian farmers increasingly rely on is irrigation.

**Fig. 1:**
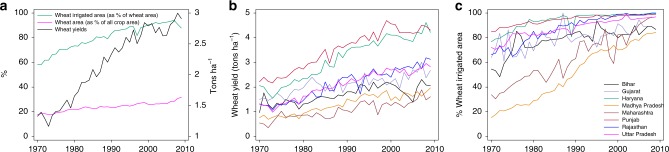
National aggregate trends in irrigation coverage, cropped area and yields, trends in yield and irrigation coverage across major wheat-producing states. Percent of wheat irrigated area is the percent of wheat cropped area that is irrigated. Percent of wheat area is the percent of total cropped area that is planted with wheat. Wheat yield is the production of wheat (in tons) per hectare. Source data are provided as a Source Data file

Irrigated crop productivity is generally found to be higher than rain-fed crop productivity^12,13^, and for decades the Indian government’s policies have promoted irrigation expansion as a method for improving agricultural growth, smoothing production risk, and alleviating rural poverty^14^. Between 1966 and 2009, while overall crop area for wheat remained stable, irrigated area saw a rapid increase (Fig. 1). This trend underscores the unique role that irrigation has played in boosting increases in yields and productivity.

Variation in irrigation availability, however, remains a deterrent to maximizing potential yield in certain regions. Recent evidence points to irrigation as one of the key factors in explaining wheat yield gaps across the western and eastern parts of the Indo-Gangetic plain^15^. Prior work has focused on irrigation as an adaptation response to rainfall changes^16–18^ but there is limited understanding of the extent to which irrigation can be used to offset temperature increases^7^. Recent work from the United States illustrates the effectiveness of irrigation in completely mitigating the harmful effects of extreme heat^19^. However, these estimates may not translate to India, where wheat is highly irrigated and where physical water availability can be considerably constrained. Some studies even show harmful temperature effects on irrigated yields in Asia^20^. Incorporating irrigation when estimating wheat yield changes to climate, therefore, provides additional information on how growing water scarcity is likely to impact future yields and can also improve our understanding of weather and water stress relationships.

Irrigation accounts for 80–90% of India’s total water demand^21,22^, with wheat production estimated to be the single main driver of the country’s increased consumptive irrigation water demand^23^. While certain regions in India have accrued large benefits from irrigation, these benefits have come at the cost of increased pressure on many irrigation water sources^16,24^. Reduced availability of water for irrigation, especially in areas where non-renewable groundwater is extracted, is likely to have impacts on future production in some states^25^ and in these regions irrigation may not be an optimal strategy. Using observed historical relationships between weather, wheat yields and irrigation across all major-wheat producing districts in India for a period of 40 years from 1970 to 2009, this paper addresses two main questions:^1^ How much has irrigation contributed to wheat yield increases over time?^2^ What role, if any, has irrigation played in reducing heat stress? We find that irrigation, on average, contributes to a 13% yield increase in the 2000s compared to a scenario without irrigation trends, with most of these increases concentrated in Madhya Pradesh (29%), Maharashtra (21%) and Bihar (15%). Irrigation reduces heat sensitivity of yields by roughly one-quarter of the impact seen in fully rainfed conditions. The rate of gain from irrigation expansion, however, has slowed in recent years. Furthering yield gains via irrigation expansion in the face of extra warming is likely to present a substantial challenge.

## Results

### Estimated impacts of irrigation on wheat yields

We use regression analysis to estimate wheat yields as a function of weather variables and irrigation to predict yield impacts under different counterfactual scenarios. We include major wheat-producing states of Punjab, Haryana, Uttar Pradesh, Bihar, Rajasthan, Madhya Pradesh, Maharashtra, and Gujarat in our analysis. Together, these states account for over 90% of national wheat production (Supplementary Fig. 1). Punjab, Uttar Pradesh and Haryana—whose share of national wheat production ranks high—saw greater improvements in yield between 1970 and 2009 relative to increases in irrigation (Fig. 1). These states were already highly irrigated in the 1970s and 1980s (70–80% and 80–90%) and by the late 2000s, had achieved close to full irrigation coverage. On the other hand, Bihar, Maharashtra, and Madhya Pradesh experienced steep increases in irrigation coverage between 1970 and 2009 compared to other states with proportionate (in the case of Bihar and Maharashtra) and less than proportionate (in the case of Madhya Pradesh) increases in yield (Supplementary Fig. 2).

In the subsequent analysis, we test whether these simple associations hold when we account for various factors that may be correlated with yields and irrigation.

Accounting for crop responses to high temperatures is important when studying weather-yield relationships in India^26^. Following current best-practice in estimating yield-weather relationships, we construct degree days using a sinusoidal interpolation of temperature exposure within each day^26,27^. The advantage of using such an approach is that it captures the full variation in extreme temperature exposure that is otherwise missed when using an alternative measure of degree days based on daily mean temperature^6^. While there is limited guidance in the literature about the appropriate degree day thresholds for wheat since these can differ depending on the region of interest as well as the method of construction^6^, temperatures >30 °C are generally found to have negative effects on yields and are considered a good measure of very hot days^6,26–28^. Thus, following prior work^26^, we control for a piece-wise linear function of both normal growing degree days (GDD) and extreme degree days (EDD) in our statistical model. We then use a log-linear model to quantify the effects of irrigation, EDD, GDD, total precipitation, and rainfall distribution on wheat yields.

Supplementary Table 1 presents summary statistics of all variables and Supplementary Fig. 3 shows trends in EDD and GDD. Weather variables tend to be correlated over time in the same location (Supplementary Table 2) and thus controlling for all of them makes it possible to estimate their separate impacts on yield. Across all models, we control for unobserved time-invariant heterogeneity at the district level using district fixed effects that accounts for soil type, socio-economic characteristics and other geographical characteristics, and year-specific shocks that are common to all districts to account for all common contemporaneous trends, such as national price changes, economic growth and population growth (see “Methods”). In addition, we account for state-specific trends that capture variation in technological progress across states and state-specific policies related to electricity, agricultural, and water subsidies. Table 1 reports the estimated regression coefficients. Columns 1 through 4 report parameter estimates for four models that sequentially include weather variables. In general, the coefficients on EDD are larger in magnitude than for GDD, suggesting that further increases in warming have a stronger impact on yields as temperatures exceed 30 °C. The harmful impact of EDD remains robust as additional weather variables are introduced. EDD coefficients are statistically significant (*p* < 0.01, two-sided *t*-test) even after inclusion of the precipitation measures and after accounting for spatial correlation (Supplementary Table 3). Similar stronger effects on yield are seen when temperatures exceed 27 °C, although the magnitude of the impact is lower than when we consider an upper threshold of 30 °C (Supplementary Fig. 4).

**Table 1:** Impact of weather and irrigation on log wheat yield

Dependent variable: Log wheat yield	(1)	(2)	(3)	(4)	(5)	(6)	(7)	(8)	(9)
EDD [30 + ] (10 days)	−0.0243***	−0.0249***	−0.0216***	−0.0220***	−0.0241***	−0.0196***	−0.0575***	−0.0449***	−0.0550***
	(0.003)	(0.004)	(0.004)	(0.004)	(0.004)	(0.002)	(0.008)	(0.008)	(0.008)
GDD [0, 30] (10 days)		−0.0001	−0.0001	−0.0002	−0.0003	0.0000	−0.0015	0.0007	−0.0016
		(0.001)	(0.001)	(0.001)	(0.001)	(0.000)	(0.001)	(0.001)	(0.001)
Precip (10 mm)			0.0010***	0.0008***	0.0008***	0.0013***	0.0012*	0.0014**	0.0053***
			(0.000)	(0.000)	(0.000)	(0.000)	(0.001)	(0.001)	(0.001)
Rainy days				0.0010**	0.0010***	0.0003	0.0015	0.0009	0.0013
				(0.000)	(0.000)	(0.000)	(0.001)	(0.001)	(0.001)
Share irrigation					0.4493***	0.5564***	0.0339	0.7749	0.1843
					(0.084)	(0.091)	(0.410)	(0.488)	(0.419)
Precip Sq.									−0.0002***
									(0.000)
EDD [30+] × Share irrigation							0.0411***	0.0316***	0.0390***
							(0.008)	(0.009)	(0.008)
GDD [0, 30] × Share irrigation							0.0011	−0.0011	0.0012
							(0.001)	(0.002)	(0.001)
Precip × Share irrigation							−0.0005	−0.0001	−0.0043***
							(0.001)	(0.001)	(0.002)
Rainy days × Share irrigation							−0.0006	−0.0009	−0.0005
							(0.002)	(0.002)	(0.002)
Precip Sq x Share irrigation									0.0002***
									(0.000)
Observations	7661	7661	7661	7661	7661	7661	7661	7661	7661
Impact of EDD under Full irrigation							−0.016	−0.013	−0.016
*P* value							0.000	0.000	0.000
Adj. Rsq	0.906	0.906	0.907	0.907	0.913	0.894	0.914	0.895	0.915
RMSE	0.159	0.159	0.158	0.158	0.153	0.169	0.151	0.168	0.151

Notes: Dependent variable is the logarithm of wheat yield. Each column represents a separate regression model. Standard errors are displayed in parentheses and are clustered at the district-level. All models include district fixed effects and state-specific linear time trends. Columns 1–5, 7 and 9 also include year fixed effects. In column 9, a quadratic precipitation term is added

Stars indicate statistical significance: *p ≤ 0.1, **p ≤ 0.05, ***p ≤ 0.01

Of the precipitation measures, we find that an increase in total precipitation in the wet season has a consistent positive effect (*p* < 0.01, two-sided *t*-test) on wheat yields. India has a monsoonal climate with a wet season that receives up to 1 m of rainfall and a dry season—during which wheat is grown—when rainfall is inadequate, and irrigation must be used^25^. The supply of rain and the amount that gets stored in reservoirs and as groundwater can, thus, impact cropping and irrigation decisions in the dry season^25^. The number of rainy days impacts the distribution of rainfall in the wet season^29^. A more even distribution of monsoon rainfall can, therefore, help retain soil moisture in the dry season. We find that additional rainy days have positive effects on wheat yields (*p* < 0.05, two-sided *t*-test).

In Columns 5 and 6 we introduce the impact of contemporaneous irrigation on wheat yields controlling for both year fixed effects and state-specific trends (columns 5) versus state-specific trends alone (column 6). We find that when the wheat-specific share of irrigation coverage increases from 0 to 1, wheat yields increase by 45–55% on average, keeping other weather variables constant. When comparing our baseline specifications with and without controlling for irrigation (columns 4 and 5 in Table 1, Supplementary Fig. 5), we find that the impact of EDD in models that account for irrigation is slightly higher than models that do not control for irrigation. Therefore, the coefficient on EDD when irrigation is not accounted for reflects a lower-bound estimate. Since the agricultural data does not parse out irrigated and rainfed yields separately, we then interact irrigation coverage with each of the weather variables to measure the extent to which irrigated yields respond to changes in each of the weather variables (Columns 7, 8 and 9 in Table 1). The statistically significant positive sign on the interaction between irrigation and EDD (*p* < 0.01, two-sided *t*-test) reflects that irrigation reduces the harmful effects of EDD. In the presence of full irrigation coverage, an additional 10 degree days of extreme heat reduces yields by 1.3–1.6%—as opposed to 4–5.7% in the absence of irrigation—across all specifications that include year fixed effects (column 7), state-specific trends (column 8) and non-linear precipitation impacts (column 9). Increases in irrigation coverage, however, are not able to completely offset these negative impacts of extreme heat.

A potential concern with using contemporaneous irrigation coverage is that irrigation is itself endogenous and responsive to changes in weather and other unobservable factors^17^, leading to a biased estimate of the effect of irrigation. To partially alleviate this concern and to remove correlations between irrigation coverage and contemporaneous weather, we also use a long-term average of irrigation share in each district that is time-invariant as well as a time-varying lagged average of irrigation share (over all previous years) in place of the contemporaneous measure (Supplementary Table 4, Supplementary Fig. 6). Regression results of all interactions between irrigation coverage and EDD are summarized in Supplementary Fig. 6. Like in our baseline model, we find that in the presence of full irrigation coverage, the negative effects of an additional 10 degree days of extreme heat are reduced and yields decrease by ~1.5% across all alternative irrigation-interaction regression models.

### Break-down of yield changes due to irrigation and weather

**Fig. 2:**
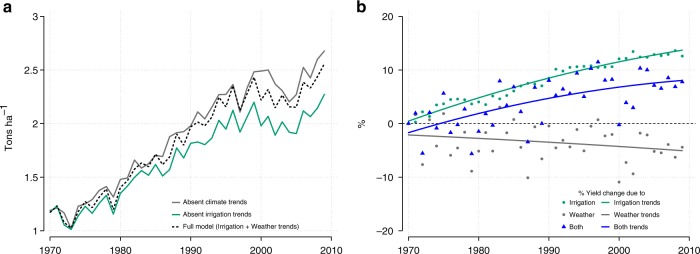
Breakdown of impacts of irrigation and weather on wheat yields over time. Full model in panel a corresponds to predicted yields using the regression model in column 5, Table 1. The three lines in panel b represent the percent of yield that is lost or gained when comparing predicted yields generated from the Full Model to predicted yields generated when (i) Irrigation is kept at average 1970–71 values (green line) (ii) All weather variables are kept at average 1970–71 values (gray line) and (iii) Irrigation as well as weather variables are kept at average 1970–71 values (blue line). Source data are provided as a Source Data file

**Fig. 3:**
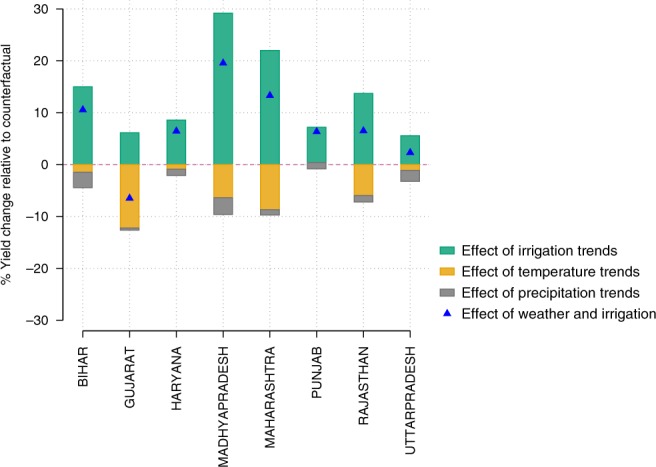
Breakdown of changes in wheat yield from irrigation and weather in 2000-09 in the major wheat-producing states. Green bars represent the percent of yield that is gained due to irrigation. Orange and gray bars represent the percent of yield that is lost due to temperature and precipitation respectively. For each state, the figure shows how far predicted yields generated from the Full Model are from predicted yields generated when (i) Irrigation is kept at average 1970–71 values; green bars (ii) Temperature variables are kept at average 1970–71 values; orange bars (iii) Precipitation variables are kept at average 1970–71 values; gray bars or (iii) Irrigation as well as weather variables are kept at average 1970–71 values; blue triangles. Source data are provided as a Source Data

To translate these impacts into relative yield changes due to irrigation and weather, we measure the difference between predicted yields using our baseline regression and predicted yields when irrigation or weather are kept at 1970 levels (Fig. 2, Supplementary Fig. 7). To assess changes at the end of the sample period, we average yield changes between 2000 and 2009 to remove concerns about fluctuations in certain years (Fig. 3).

Over time, the benefits accrued from irrigation have outpaced the costs imposed by weather. We find that the amount of yield that is gained due to irrigation trends upwards over the entire time-period (Fig. 2a) from an average 6% gain in the 1980s to a 13% gain in the 2000s (green line in Fig. 2b). We also find that wheat yields are around 5% lower than they would have been absent weather changes towards the end of the study period (Fig. 2b). The amount of warming since 1970 has, thus, been enough to reduce annual yields by nearly half of the gains seen from irrigation over the same time period. On average, the amount of gain from irrigation expansion more than compensates for the losses from warming over the period of analysis. Other studies have similarly found that irrigation access remains a major contributor to yield variability in India^15^ and globally^30,31^. The finding that warming has suppressed yields by 5% is also in line with previous estimates for this region^5^. These average national gains and losses, however, mask considerable spatial heterogeneity (Supplementary Fig. 7).

Relative yield changes across the major wheat-producing states are summarized in Fig. 3. Across the majority of states, yield gains due to irrigation (green bars in Fig. 3) outweigh yield losses due to changes in temperature and precipitation (orange and gray bars in Fig. 3). Bihar, Madhya Pradesh and Maharashtra, although producing a lower percentage of India’s wheat (Supplementary Fig. 1), show the greatest relative yield increases by the end of the period. A majority of this increase is attributed to irrigation. Wheat yields are 15, 29, and 21% higher in Bihar, Madhya Pradesh, and Maharashtra at the end of the sample period relative to a baseline irrigation scenario (Fig. 3).

On the other hand, in high wheat production states such as Punjab, Haryana and Uttar Pradesh that already experienced high levels of irrigation in the early years of the analysis, any further gain from irrigation diminishes over time (Supplementary Fig. 8) and is far more limited by the 2000s (Fig. 3). We find that wheat yields are only 5–8% higher at the end of the sample period compared to a baseline irrigation scenario (Fig. 3). It is possible that other factors like minimum support price policies^32^ and subsidies for electricity^33^ have played a substantial role in influencing wheat yield gains in addition to irrigation in these states. Moreover, groundwater irrigation is widespread in these states and the drawdown of water is fast becoming a binding constraint on agricultural productivity^16,24,25^. We also find that these states show little to no impact of weather on relative yield changes, reflecting a combination of smaller weather trends in these states and lower sensitivity to weather owing to the predominance of irrigation.

The state of Gujarat is the only outlier where we find that yield losses due to weather changes, out-measure any yield gains from irrigation (Fig. 3 and Supplementary Fig. 8). Wheat yields by the 2000s are ~12% lower than they would have been absent any weather changes, and 6% lower even after accounting for any gains from irrigation (Fig. 3*)*. Trends in EDD and GDD contribute to the majority of this loss.

In addition to irrigation, multiple institutional, political and economic forces have contributed to yield gains. At the heart of farming activity in many regions of India are state subsidies in their various guises. Both input subsidies and output subsidies (guaranteed procurement at set prices that feeds the country’s Public Distribution System) influence cropping and investment decisions of farmers. Subsidies for electricity—the key input used for groundwater extraction—amount to 85% of the average cost of supply and play a critical role in enabling groundwater extraction^33^. Free or flatly tariffed electricity provision have led to an increase in the value of irrigation-intensive crops, and hence the area on which these crops are grown^33^. Output subsidies such as minimum support prices for rice and wheat, and their procurement by government agencies directly impact agricultural markets and the price that farmers receive. This, in turn, can skew cropping decisions in favor of these crops even in areas not conducive for their growth^32^. The impact of these policy instruments, however, are far from uniform, and large variations exist across states and regions (Supplementary Table 5)

While the western and north-western states rely on well-developed groundwater and canal systems, along with highly subsidized electricity, states in the east have access to less developed canal systems and unsubsidized diesel pumps^14^. Procurement of wheat is the highest in the model Green Revolution states of Punjab and Haryana, where irrigated wheat productions systems now occur at large scales (32, Supplementary Table 5). In other states, government procurement agencies are limited and absent, or are only recently expanding their activities (for example, Madhya Pradesh)^34^. Understanding the relative contribution of all these additional components to wheat yield gains can help uncover potential trade-offs between energy, water and food security policies, and remains an important area of future work.

## Discussion

Irrigated wheat production is likely to be subject to hotter temperatures than those observed in the historical data. We find that irrigation can alleviate the harmful effects of extreme heat to the extent that irrigation can be expanded. The largest increases in yields due to irrigation are seen in Madhya Pradesh, Maharashtra and Bihar. It is possible that in these states yields could be raised even higher with improvements in irrigation access that the states in the west and north-west have benefited from in the past. However, any increase in irrigation access should also be accompanied by sustainability considerations to avoid groundwater depletion that high-irrigated states of Punjab and Haryana are now facing^16,24,25^. On the other hand, the contribution of irrigation to yield increases is the lowest in Gujarat where temperature trends have outpaced the benefits of irrigation. As a result, it is possible that irrigation will no longer be able to buffer the harmful effects of extreme heat in the future. The contribution of irrigation also remains limited in already high-irrigated states of Punjab and Haryana. Further use of irrigation as an adaptation mechanism in these regions may, therefore, not be feasible as irrigation reaches full capacity. Even as the role of irrigation diminishes, it is possible that other factors outside of irrigation such as procurement of wheat at minimum support prices will continue to govern wheat production and contribute to wheat yield gains in these states (Supplementary Table 5). More work is required to fully understand the physical constraints on irrigation as temperatures rise, and the optimal level of irrigation capacity needed in the long term.

The future of Indian wheat productivity will be determined by many factors, including technological innovation, global markets, and government policies. Although irrigation and weather are only two factors, they have each played an important role in shaping yield trajectories over recent decades (Figs. 2 and 3). Even though irrigation has accrued net benefits, on average, and gains have increased from 3% in the 1970s to 13% in the 2000s, the rate of gain from irrigation expansion has been slowing down. The trend in irrigation benefits between 1970 and 2000 is statistically different from the trend in the 2000s (*p* < 0.001, two-sided *t*-test). On average, gains from irrigation increase by 7.5% per year up to 2000 but subsequently increase at a diminished rate of 0.6% per year (green line in Fig. 2b; the linear and quadratic trend coefficients are jointly significant, *p* < 0.001, two-sided *t*-test). The impacts of weather changes, on the other hand, appear more linear throughout the study period, with effects of cumulative warming since 1970 equal to ~5% yield loss by the 2000s (gray line in Fig. 2b). This indicates that despite increased irrigation and the associated reduction in temperature sensitivity, the pace of temperature increase has been sufficient to sustain linear growth in heat impacts. Although irrigation is an effective adaptation to warming, it is unlikely that any further increases will be large enough to substantially slow down the impacts of warming across all states. Instead, the inability for irrigation to increase much more than its current extent in certain states means that climate impacts may accelerate in the absence of other adaptation strategies, since increased irrigation will no longer be cushioning the impact of warming.

## Methods

### Data

District-wise agricultural data for India from 1970 to 2009 are from the Village Dynamics in South Asia (VDSA) database. The wet season coincides with the timing of the summer monsoon, which spans approximately June through September. The dry season when wheat is grown spans approximately October through February. We constructed district-level weather data by averaging gridded temperature and precipitation data from the Indian Meteorological Department over the growing season and using maps corresponding to 1966 district boundaries to aggregate to the district. Districts and states that split after 1966 are considered together to allow comparability over time. Additional information is provided in Supplementary Note 1

### Estimating impacts of irrigation on wheat yields

To estimate the relationship between weather variables, irrigation and wheat yields, we use a multivariate log-linear regression shown in Eq. (1)

1 $$\log \mathrm{Yield}_{\mathrm{d}t} =	 \, \alpha _d + \lambda _{s,t} + \rho _t + \theta _1\mathrm{GDD}_{\mathrm{d}t} + \theta _2\mathrm{EDD}_{\mathrm{d}t} + \theta _3{\boldsymbol{P}}_{{{\mathbf{d}t}}} + \beta _1\mathrm{GDD}_{\mathrm{d}t}I_{\mathrm{d}t} \\ 	+ \, \beta _2\mathrm{EDD}_{\mathrm{d}t}I_{\mathrm{d}t} + \beta _3{\boldsymbol{P}}_{{{\mathbf{d}t}}}I_{\mathrm{d}t} + \gamma I_{\mathrm{d}t} + {\epsilon }_{\mathrm{d}t}$$

Yield_d*t*_ is wheat yield in district *d* and year *t*. Using logged yield allows temperature and precipitation to affect yields proportionally. EDD_d*t*_ captures cumulative EDD, i.e., the number of days in the growing season with temperatures above 30 °C and GDD_d*t*_ measures cumulative GDD between the lower threshold of 0 °C and upper threshold of 30 °C. Although the sample average of cumulative EDD above 30 °C is low (Supplementary Table 1), more than 90% of the observations experience nonzero EDD. In alternative specifications, we use an upper threshold of 27 °C and a lower threshold of 5 °C^11^.

We also include ***P***_**d*t***_, a vector that accounts for a quadratic in total precipitation in the preceding monsoon season as well as a measure of the distribution of rainfall. We include the latter since decreases in the number of rainy days have been shown to have negative impacts that are large enough to overturn the benefits of increased total precipitation^28^. Following^28^, the distribution of rainfall is measured by the number of rainy days, i.e., days with precipitation > 0.1 mm. Correlations between the weather variables are shown in Supplementary Table 2.

Following standard practices in the literature that use statistical analysis to estimate yield-weather relationships, the model includes district time-invariant controls, year fixed effects and state trends. Accounting for these controls is necessary to isolate the impact of exogenous changes in temperature and precipitation on wheat yields. Similar approaches have been used in previous studies on crop yields in India^7,29,35^ and other countries^27,36^. A concern with using fixed effects, however, is that these controls can absorb much of the variation in weather. Supplementary Table 6 provides the *R*-square and standard deviation of the residual variation in weather that remains unabsorbed by different sets of fixed effects. For instance, including only year fixed effects preserves a significant amount of variation, but including district fixed effects the remaining variation decreases substantially, suggesting that spatial differences account for a majority of the variation. There is no further reduction in the variation when state-specific time trends are included. To account for within-district clustering of errors and arbitrary correlation of observations across time, cluster-robust standard errors are used. To account for spatial autocorrelation, we also use Driscoll and Kraay standard errors^37^, which are robust to spatial and temporal dependence (up to two lags of autocorrelation) (See Supplementary Table 3). All the yield regressions are area-weighted by 1970–2009 average wheat area.

To measure the change in sensitivity of yields to weather due to irrigation coverage, we include a time-dependent share of wheat-specific irrigated area, *I*_*dt*_, and its interactions with the weather variables. Since our data does not provide information on irrigated and unirrigated yields separately, we are unable to directly estimate the impact of weather on irrigated and unirrigated yields. Instead, the data provides information on the share of wheat cropped area that is irrigated for each district and year which we use as our measure of wheat irrigation. The coefficients *β*_1_, *β*_2_, *β*_3_ measure how full irrigation coverage can change the sensitivity of wheat yields to weather impacts.

### Contribution of irrigation and weather to yield changes

The estimates from our statistical model are then used to measure the contribution of irrigation and weather to yield changes over time. For this purpose, we calculate the percent change between predicted yields from our main regression model and predicted yields from a counterfactual scenario. We consider three counterfactual scenarios: in the first scenario, we keep irrigation at 1970 levels (averaged over 1970–71), in the second scenario, we hold temperature and precipitation at 1970 levels (averaged over 1970–71) and in the third scenario we only include common trends and other state-specific trends, holding irrigation, temperature and precipitation at 1970 levels. To measure overall impacts of irrigation and weather for each of the major wheat-producing states, we sum the district-wise percent changes.

### Caveats

Our analysis has several caveats. Our baseline irrigation scenario is simple and meant to highlight the extent to which irrigation has contributed to wheat yield gains over time. It does not consider the direct impacts of climate change on irrigation water and groundwater recharge^25,38^ which would be needed to fully quantify the additional amount of irrigation capacity needed and the possibility of further groundwater depletion. Our results depend on the model specification and the type of counterfactual scenario we use. Since we include year fixed effects and state-linear trends to predict yields in the counterfactual scenarios we assume that these are independent of irrigation and weather trends. Moreover, like other statistical models, we are unable to account for feedback between intense land-cover change and cooling temperature extremes^39^ that can influence the relationship between extreme heat and wheat yields.

### Reporting summary

Further information on research design is available in the Nature Research Reporting Summary linked to this article.

## Supplementary information

Supplementary Information

Reporting Summary

Source Data

## Data Availability

The primary agricultural data used in this analysis are publicly available from the International Crop Research Institute for the Semi-Arid Tropics (ICRISAT) and their Village Dynamics in South Asia (VDSA) database (http://vdsa.icrisat.ac.in/vdsa-database.aspx). Observed temperature and precipitation data were acquired from the Indian Meteorological Department (http://www.imdpune.gov.in/ndc_new/Request.html). The data that support the findings of this study are available from the corresponding author upon request. The source data underlying Figs. 1–3 are provided as a Source Data file.

## References

[1] Ministry of Agriculture. Agricultural Statistics at a Glance 2014, Directorate of Economics and Statistics, Government of India (2014)

[2] Food and Agriculture Organization of the United Nations, Food and Agriculture Organization of the United Nations Statistical Database (FAOSTAT); faostat.fao.org.

[3] Shiferaw B (2013). Crops that feed the world 10. Past successes and future challenges to the role played by wheat in global food security. Food Sec..

[4] Singh, R. B., Kumar, P., Woodhead, T. *Smallholder Farmers in India: Food Security and Agricultural Policy*. (FAO Regional Office for Asia and the Pacific, Bangkok, Thailand, 2002).

[5] Lobell DB, Schlenker W, Costa-Roberts J (2011). Climate trends and global crop production since 1980. Science.

[6] Tack J, Barkley A, Nalley LL (2015). Effect of warming temperatures on US wheat yields. Proc. Natl Acad. Sci. USA.

[7] Taraz V (2018). Can farmers adapt to higher temperatures? Evidence from India. World Dev..

[8] Asseng S (2014). Rising temperatures reduce global wheat production. Nat. Clim. Change.

[9] Lobell DB (2008). Prioritizing climate change adaptation needs for food security in 2030. Science.

[10] Intergovernmental Panel on Climate Change. (2014). Climate Change 2013 The Physical Science Basis Working Group I Contribution to the Fifth Assessment Report of the Intergovernmental Panel on Climate Change.

[11] Gupta R, Somanathan E, Dey S (2017). Global warming and local air pollution have reduced wheat yields in India. Clim. Change.

[12] Fischer G, Tubiello FN, Van Velthuizen H, Wiberg DA (2007). Climate change impacts on irrigation water requirements: effects of mitigation, 1990-2080. Technol. Forecast. Soc. Change.

[13] Bruinsma, J. et al. The resource outlook to 2050: by how much do land, water and crop yields need to increase by 2050? in How to feed the World in 2050. Proceedings of a Technical Meeting of Experts, Rome, Italy, 24–26 June 2009, 1–33 (Food and Agriculture Organization of the United Nations (FAO), 2009)).

[14] Shah T (2010). Taming the Anarchy: Groundwater Governance in South Asia.

[15] Jain M (2017). Using satellite data to identify the causes of and potential solutions for yield gaps in India’s Wheat Belt. Environ. Res. Lett..

[16] Fishman R (2018). Groundwater depletion limits the scope for adaptation to increased rainfall variability in India. Clim. Change.

[17] Taraz V (2017). Adaptation to climate change: Historical evidence from the Indian monsoon. Environ. Dev. Econ..

[18] Siegfried T (2010). Modeling irrigated area to increase water, energy, and food security in semiarid India. Weather, Clim., Soc..

[19] Tack J, Barkley A, Hendricks N (2017). Irrigation offsets wheat yield reductions from warming temperatures. Environ. Res. Lett..

[20] Welch JR (2010). Rice yields in tropical/subtropical Asia exhibit large but opposing sensitivities to minimum and maximum temperatures. Proc. Natl Acad. Sci. USA.

[21] Wada Y (2013). Multimodel projections and uncertainties of irrigation water demand under climate change. Geophys. Res. Lett..

[22] Famiglietti JS (2014). The global groundwater crisis. Nat. Clim. Chang..

[23] Davis KF (2018). Alternative cereals can improve water use and nutrient supply in India. Sci. Adv..

[24] Rodell M, Velicogna I, Famiglietti JS (2009). Satellite-based estimates of groundwater depletion in India. Nature.

[25] Zaveri E (2016). Invisible water, visible impact: groundwater use and Indian agriculture under climate change. Environ. Res Lett..

[26] Lobell DB, Sibley A, Ortiz-Monasterio JI (2012). Extreme heat effects on wheat senescence in India. Nat. Clim. Change.

[27] Schlenker W, Roberts MJ (2009). Nonlinear temperature effects indicate severe damages to U.S. crop yields under climate change. Proc. Natl Acad. Sci. USA.

[28] Lobell DB, Bänziger M, Magorokosho C, Vivek B (2011). Nonlinear heat effects on African maize as evidenced by historical yield trials. Nat. Clim. Change.

[29] Fishman R (2016). More uneven distributions overturn benefits of higher precipitation for crop yields. Environ. Res. Lett..

[30] Ray DK, Gerber JS, MacDonald GK, West PC (2015). Climate variation explains a third of global crop yield variability. Nat. Commun..

[31] Mueller N (2012). Closing yield gaps through nutrient and water management. Nature.

[32] Chatterjee S, Kapur D (2017). Six puzzles in indian agriculture. India Policy Forum 2016.

[33] Badiani R, Jessoe KK, Plant S (2012). Development and the environment: the implications of agricultural electricity subsidies in India. J. Environ. Dev..

[34] Krishnamurthy, M. States of wheat: the changing dynamics of public procurement in Madhya Pradesh. *Economic Political Weekly*, **47**, 72–83 (2012)

[35] Auffhammer M, Ramanathan V, Vincent J (2012). Climate change, the monsoon, and rice yield in India. Clim. Change.

[36] Schlenker W, Lobell DB (2010). Robust negative impacts of climate change on African agriculture. Environ. Res. Lett..

[37] Driscoll JC, Kraay AC (1998). Consistent covariance matrix estimation with spatially dependent panel data. Rev. Econ. Stat..

[38] Elliott J (2014). Constraints and potentials of future irrigation water availability on agricultural production under climate change. Proc. Natl Acad. Sci. USA.

[39] Mueller ND (2017). Global relationships between cropland intensification and summer temperature extremes over the last 50 years. J. Clim..

