# Supplementary material for: The role of irrigation in changing wheat yields and heat sensitivity in India
Source: Nat Commun. 2019 Sep 12;10:4144. doi: 10.1038/s41467-019-12183-9 (PMC6742628; doi:10.1038/s41467-019-12183-9)
Supplement: Supplementary file 1 — Supplementary Information [file 41467_2019_12183_MOESM1_ESM.docx]

**Supplementary Information for**

The role of irrigation in changing wheat yields and heat sensitivity in India

Zaveri et al. Nature Communications, 2019

**Supplementary Information**

**Supplementary Note 1**

Data Sources

The historical agricultural data used in our analysis was acquired from the International Crop Research Institute for the Semi-Arid Tropics (ICRISAT) and their Village Dynamics in South Asia (VDSA) database, which collates data from State Directories of Agriculture, State Bureaus of Economics and Statistics, State Planning Departments, various Agricultural Censuses, and government reports. The dataset includes district-level data across all major agricultural states in India. We use data from 1970-2009 in our analysis since a majority of observations for the major wheat-producing states are available in this time-period. A district is an administrative unit under the Indian state that is the lowest level of disaggregation for which agricultural data are uniformly available across India. The average district area of 5000 sq. km. supports an average population of two million. This is roughly twice the average area of a U.S. county (2,584 sq. km.), and nearly 18 times greater than the average population of a U.S. county (100,000). Observed temperature and precipitation data were acquired from the Indian Meteorological Department at a spatial resolution of 1° x 1° (temperature) and 0.25° x 0.25° (precipitation). We re-scale the gridded weather data to the district level by taking an area-weighted average of grid values in each district, using maps corresponding to 1966 district boundaries.

**Supplementary Figures**

Supplementary Figure 1. Contribution of each state to national wheat production


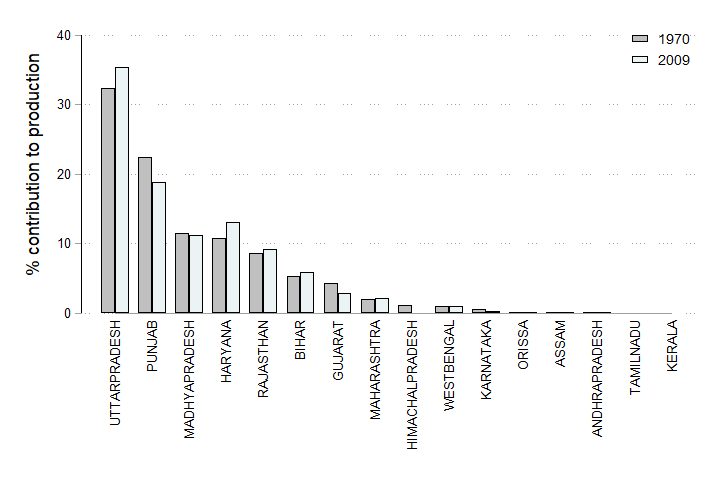


Note: Percent contribution of each state to national wheat production for the first and last year of the sample period. The first 8 states from the left are classified as major wheat-producing states

Supplementary Figure 2. Relative yield and irrigation change between the 1970 and 2009 across major wheat-producing states


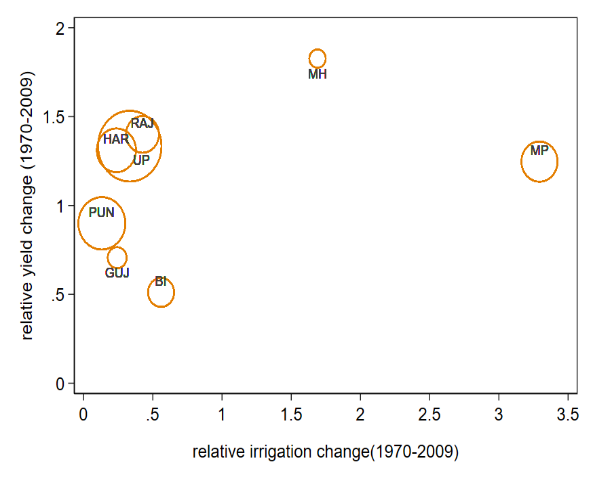


Note: Relative yield change is defined as the ratio of average yield in 2005-09 over average yield in 1970-74 subtracted by 1. Relative irrigation change is similarly defined. Size of the bubble indicates each state’s contribution to national wheat production in 2009. States are defined as follows: BI: Bihar, GUJ: Gujarat, HAR: Haryana, MH: Maharashtra, MP: Madhya Pradesh, PUN: Punjab, RAJ: Rajasthan, UP: Uttar Pradesh

Supplementary Figure 3. National and state-wise trends in EDD[30+] and GDD[0,30] across major wheat-producing states


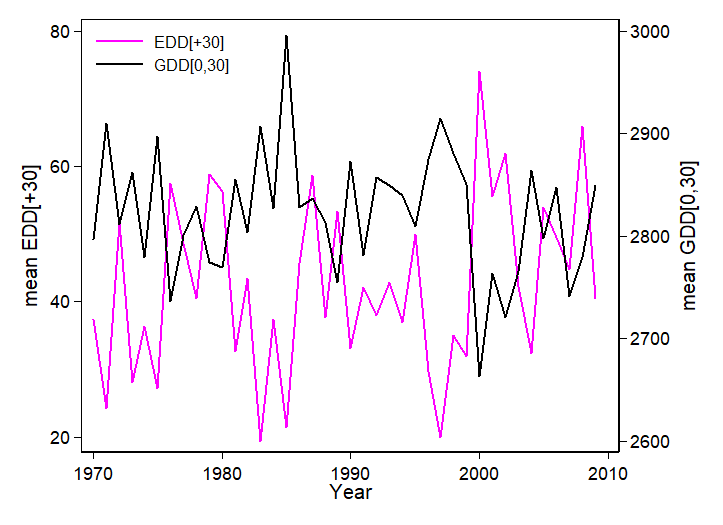

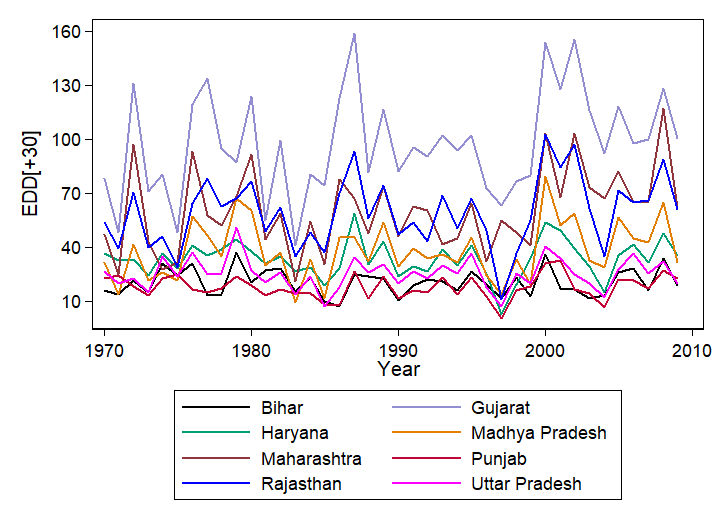

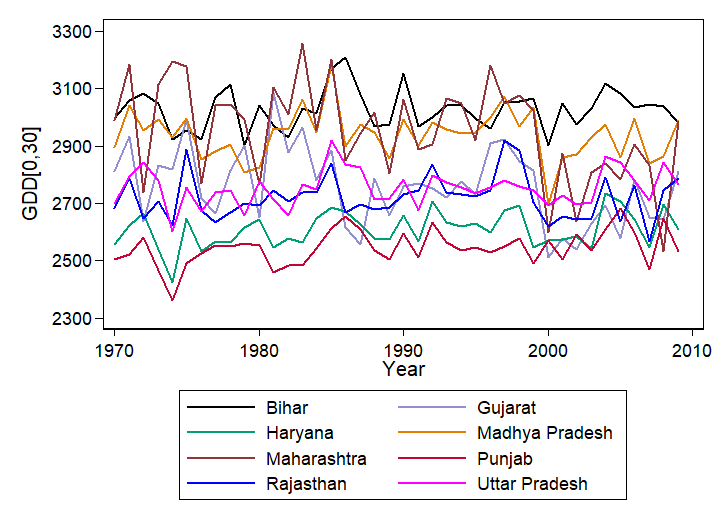


Supplementary Figure 4. Impacts of EDD, GDD, precipitation and number of rainy days on log wheat yield across two regression models using different temperature thresholds.


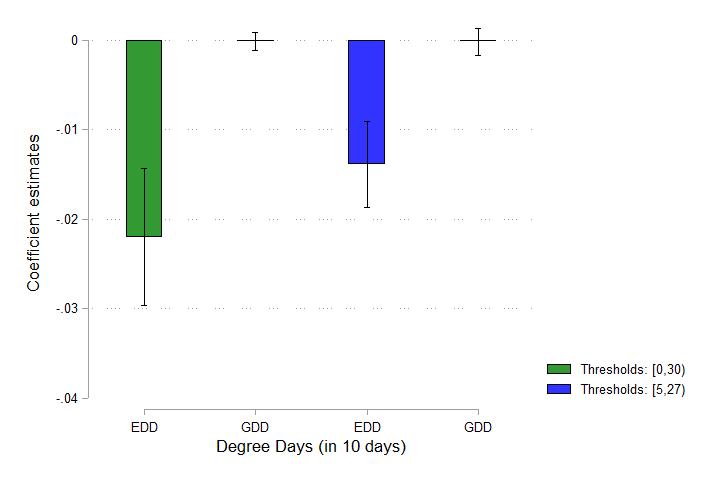

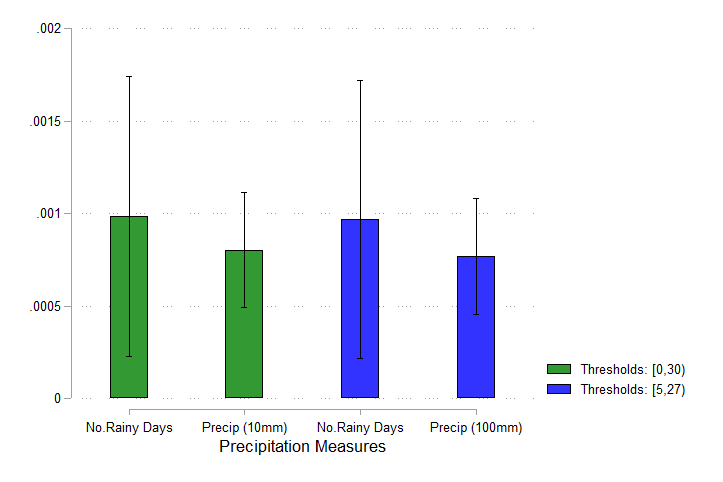


Note: Thresholds: [0,30) corresponds to degree day variables constructed using a lower threshold of 0 and upper threshold of 30. Thresholds: [5, 27) is similarly defined. On average, an increase of one rainy day increases total precipitation by 15mm. Error bars indicate 95% confidence intervals

Supplementary Figure 5. Coefficient estimates for EDD[30+] and GDD[0,30] with and without irrigation controls.


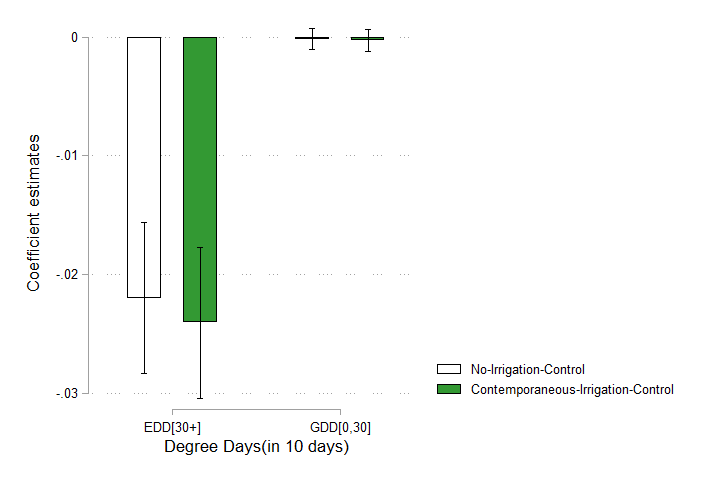


Note: Estimates correspond to columns 4 and 5 in Table 1. Error bars indicate 95% confidence intervals

Supplementary Figure 6: Impact of EDD [30+] on log wheat yield across shares of irrigation coverage.


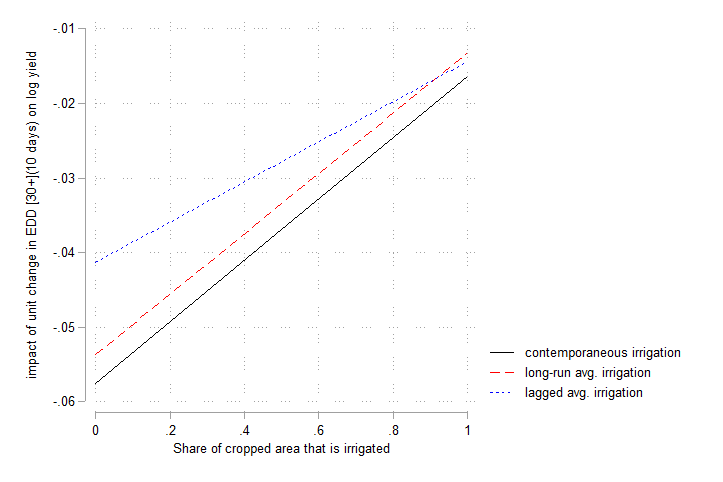


Note. Each line is a separate regression model showing the impact of EDD [30+] under no irrigation (share of irrigation is 0) and full irrigation (share of irrigation is 1). The black, red and blue lines correspond to column 8 in Table 1, and columns 1 and 4 in Supplementary Table 6 respectively where the interaction between EDD [30+] and irrigation coverage reflects the impact of EDD[30+] under full irrigation. Further details on the regression models are explained in Table 1 and Table S6. Standard errors are not reported for clarity.

Supplementary Figure 7. Actual versus predicted wheat yields
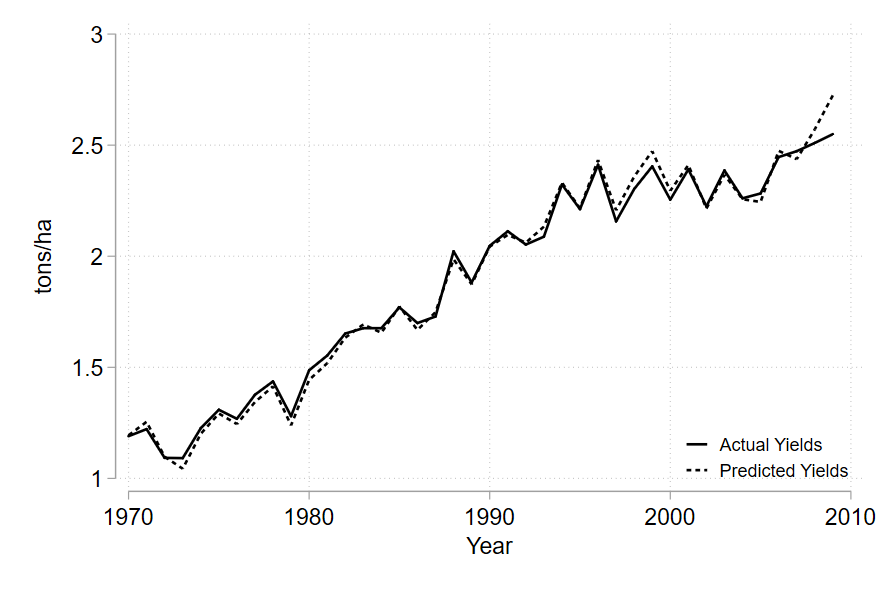


Note. Predicted yield corresponds to predicted yields using the regression model in column 5, Table 1.


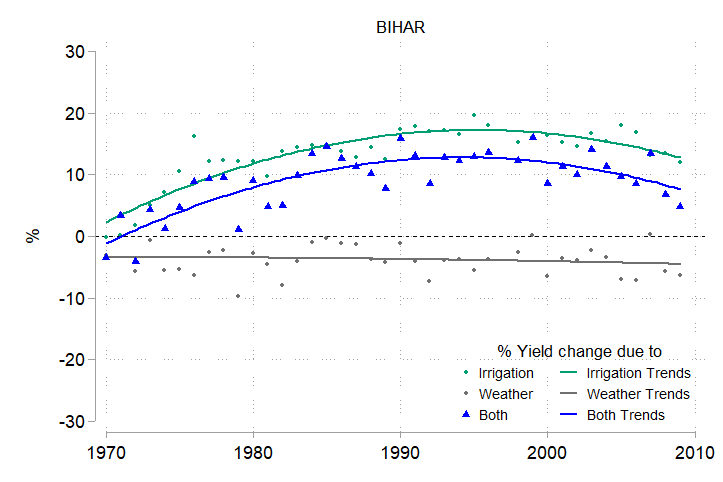
 Supplementary Figure 8. State-wise breakdown of changes in wheat yield from irrigation and weather


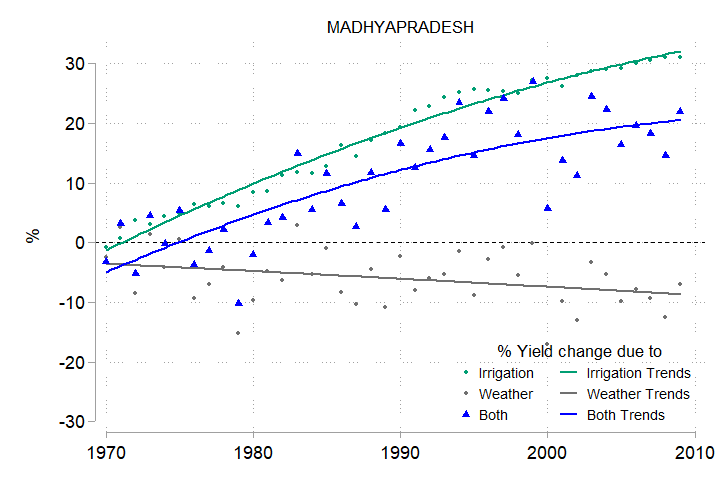

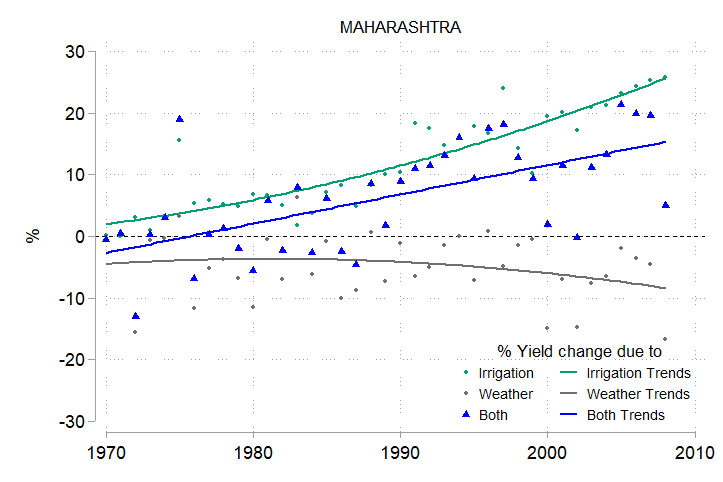

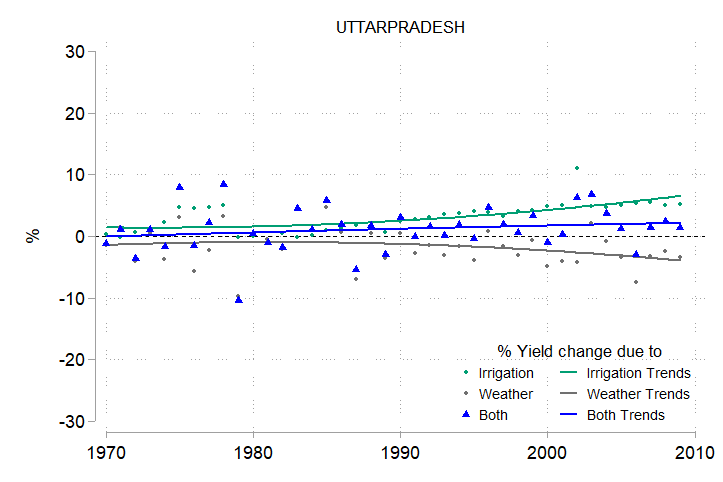

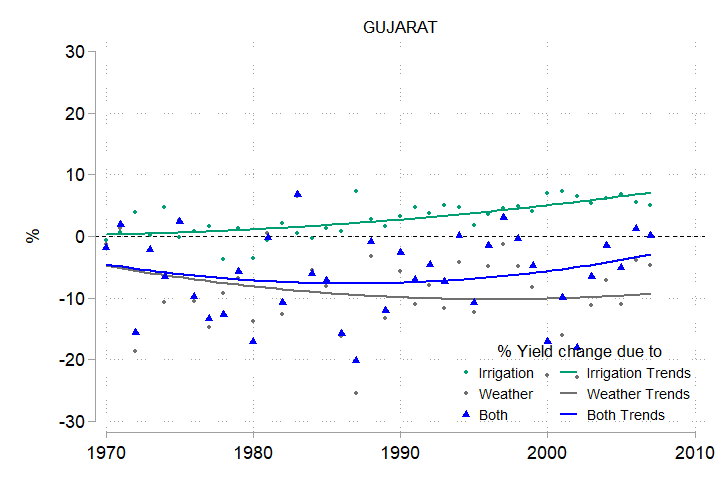

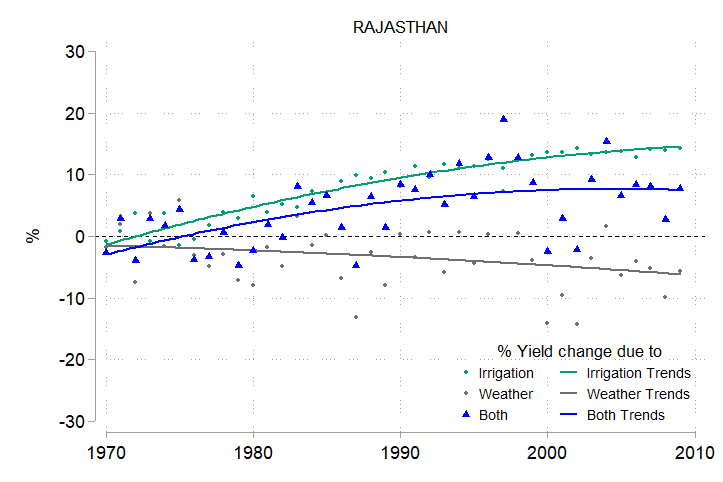

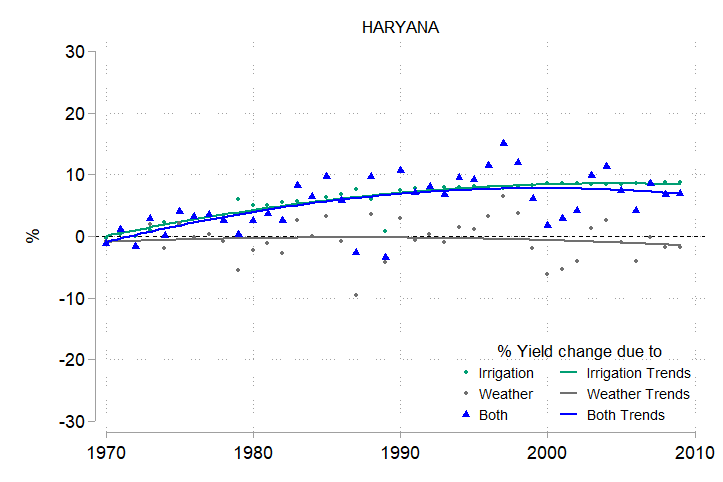

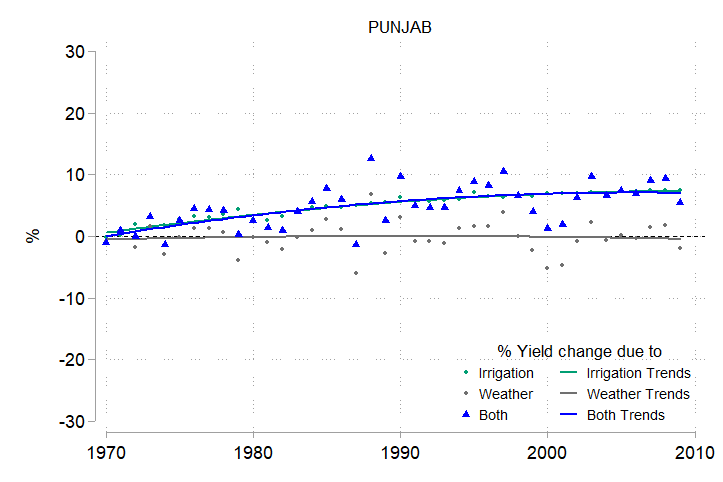


Note: Breakdown of impacts of irrigation and weather on wheat yields over time. The three lines represent the percent of yield that is lost or gained when comparing predicted yields generated from the Full Model to predicted yields generated when (i) Irrigation is kept at average 1970-71 values (green line) (ii) All weather variables are kept at average 1970-71 values (grey line) and (iii) Irrigation as well as weather variables are kept at average 1970-71 values (dotted blue line).

**Supplementary Tables**

Supplementary Table 1. Summary Statistics

|  |  |  |  |  |  |
| --- | --- | --- | --- | --- | --- |
| Variable | Obs | Mean | Std. Dev. | Min | Max |
| GDD[0,30] (10 days) | 7,661 | 282.1381 | 19.87565 | 215.0321 | 354.4408 |
| EDD[30+] (10 days) | 7,661 | 4.20518 | 3.079112 | 0 | 19.63032 |
| Monsoon Rain (10 mm) | 7,661 | 79.84018 | 35.91794 | 1.314312 | 532.6444 |
| No. of Rainy Days | 7,661 | 25.73698 | 15.28705 | 0 | 108 |
| Share Wheat irrigation | 7,661 | 0.7512673 | 0.281415 | 0 | 1 |
| Wheat Yield (tons per ha) | 7,661 | 1.822996 | 0.9025147 | 0.0743243 | 6.323529 |

|  |  |  |  |  |  |
| --- | --- | --- | --- | --- | --- |
|  | Monsoon | No. of Rainy Days | GDD [0,30] | EDD [+30] | Share irrigated area |
| Monsoon Rain | 1 |  |  |  |  |
| No. of Rainy Days | 0.7679* | 1 |  |  |  |
| GDD [0,30] | 0.3351* | 0.2389* | 1 |  |  |
| EDD [+30] | -0.1644* | -0.2291* | -0.2788* | 1 |  |
| Share irrigated area | -0.3175* | -0.3695* | -0.2700* | 0.1656* | 1 |
|  |  |  |  |  |  |

Supplementary Table 2. Correlation of Weather Variables
Note: Correlation of main variables in Table 1. Statistical significance is given by * p<0.05.

Supplementary Table 3 : Accounting for spatial correlation

|  | (1) | (2) | (3) | (4) | (5) | (6) | (7) | (8) | (9) |
| --- | --- | --- | --- | --- | --- | --- | --- | --- | --- |
|  | Dependent variable: Log wheat yield | | | | | | | | |
| EDD [30+] (10 days) | -0.0243*** | -0.0249*** | -0.0216*** | -0.0220*** | -0.0241*** | -0.0196** | -0.0575*** | -0.0449*** | -0.0550*** |
|  | (0.005) | (0.006) | (0.005) | (0.005) | (0.005) | (0.008) | (0.008) | (0.010) | (0.008) |
| GDD [0, 30] ( 10 days) |  | -0.0001 | -0.0001 | -0.0002 | -0.0003 | 0.0000 | -0.0015 | 0.0007 | -0.0016 |
|  |  | (0.001) | (0.001) | (0.001) | (0.001) | (0.001) | (0.002) | (0.002) | (0.002) |
| Precip (10mm) |  |  | 0.0010*** | 0.0008*** | 0.0008*** | 0.0013*** | 0.0012 | 0.0014* | 0.0053*** |
|  |  |  | (0.000) | (0.000) | (0.000) | (0.000) | (0.001) | (0.001) | (0.001) |
| Rainy Days |  |  |  | 0.0010 | 0.0010 | 0.0003 | 0.0015 | 0.0009 | 0.0013 |
|  |  |  |  | (0.001) | (0.001) | (0.001) | (0.001) | (0.002) | (0.001) |
| Share Irrigation |  |  |  |  | 0.4493*** | 0.5564*** | 0.0339 | 0.7749 | 0.1843 |
|  |  |  |  |  | (0.039) | (0.040) | (0.365) | (0.497) | (0.383) |
| Precip Sq. |  |  |  |  |  |  |  |  | -0.0002*** |
|  |  |  |  |  |  |  |  |  | (0.000) |
| EDD [30+] x Share Irrigation |  |  |  |  |  |  | 0.0411*** | 0.0316*** | 0.0390*** |
|  |  |  |  |  |  |  | (0.010) | (0.009) | (0.010) |
| GDD [0, 30] x Share Irrigation |  |  |  |  |  |  | 0.0011 | -0.0011 | 0.0012 |
|  |  |  |  |  |  |  | (0.001) | (0.002) | (0.001) |
| Precip x Share Irrigation |  |  |  |  |  |  | -0.0005 | -0.0001 | -0.0043*** |
|  |  |  |  |  |  |  | (0.001) | (0.001) | (0.001) |
| Rainy Days x Share Irrigation |  |  |  |  |  |  | -0.0006 | -0.0009 | -0.0005 |
|  |  |  |  |  |  |  | (0.002) | (0.002) | (0.002) |
| Precip Sq x Share Irrigation |  |  |  |  |  |  |  |  | 0.0002*** |
|  |  |  |  |  |  |  |  |  | (0.000) |
| Observations | 7661 | 7661 | 7661 | 7661 | 7661 | 7661 | 7661 | 7661 | 7661 |
| Impact of EDD under Full Irrigation |  |  |  |  |  |  | -0.016 | -0.013 | -0.016 |
| P value |  |  |  |  |  |  | 0.010 | 0.090 | 0.020 |

Notes: Dependent variable: the logarithm of wheat yield. Each column represents a separate regression model. Driscoll-Kraay standard errors that correct for spatial correlation and temporal dependence are displayed in parentheses. Stars indicate statistical significance: * *p* ≤ 0*.*1, ** *p* ≤ 0*.*05, ****p* ≤ 0*.*01.

All models include district fixed effects and state specific linear time trends. Columns 1-5, 7 and 9 also include year fixed effects. In column 9, a quadratic precipitation term is added.

Supplementary Table 4: Alternative irrigation coverage measures

|  | (1) | (2) | (3) | (4) | (5) | (6) |
| --- | --- | --- | --- | --- | --- | --- |
|  | Dependent variable: Log wheat yield | | | | | |
| EDD [30+] (10 days) | -0.0551*** | -0.0628*** | -0.0508*** | -0.0420*** | -0.0336*** | -0.0393*** |
|  | (0.013) | (0.013) | (0.013) | (0.010) | (0.009) | (0.010) |
| GDD [0, 30] (10 days) | -0.0026 | -0.0026 | -0.0025 | -0.0013 | 0.0008 | -0.0012 |
|  | (0.002) | (0.002) | (0.002) | (0.001) | (0.001) | (0.001) |
| Precip (10mm) | 0.0021*** | 0.0022*** | 0.0070*** | 0.0018*** | 0.0020*** | 0.0058*** |
|  | (0.001) | (0.001) | (0.002) | (0.000) | (0.001) | (0.001) |
| Rainy Days | 0.0026* | 0.0017 | 0.0021 | 0.0012 | 0.0004 | 0.0009 |
|  | (0.001) | (0.001) | (0.001) | (0.001) | (0.001) | (0.001) |
| EDD [30+] x Share Irrigation | 0.0413*** | 0.0554*** | 0.0375*** | 0.0269*** | 0.0226** | 0.0241** |
|  | (0.014) | (0.015) | (0.013) | (0.010) | (0.011) | (0.010) |
| GDD [0, 30] x Share Irrigation | 0.0027 | 0.0029 | 0.0026 | 0.0009 | -0.0014 | 0.0008 |
|  | (0.002) | (0.002) | (0.002) | (0.002) | (0.002) | (0.002) |
| Precip x Share Irrigation | -0.0018** | -0.0011 | -0.0068*** | -0.0016** | -0.0009 | -0.0062*** |
|  | (0.001) | (0.001) | (0.002) | (0.001) | (0.001) | (0.002) |
| Rainy Days x Share Irrigation | -0.0021 | -0.0019 | -0.0015 | -0.0000 | -0.0001 | 0.0002 |
|  | (0.002) | (0.002) | (0.002) | (0.001) | (0.001) | (0.001) |
| Precip Sq. |  |  | -0.0002*** |  |  | -0.0002*** |
|  |  |  | (0.000) |  |  | (0.000) |
| Precip Sq x Share Irrigation |  |  | 0.0002*** |  |  | 0.0002*** |
|  |  |  | (0.000) |  |  | (0.000) |
| Share Irrigation |  |  |  | 0.5887 | 1.3667** | 0.8277 |
|  |  |  |  | (0.497) | (0.532) | (0.503) |
| Observations | 7661 | 7661 | 7661 | 7463 | 7463 | 7463 |
| Impact of EDD under Full Irrigation | -0.014 | -0.007 | -0.013 | -0.015 | -0.011 | -0.015 |
| P value | 0.000 | 0.010 | 0.000 | 0.000 | 0.000 | 0.000 |
| Adj. Rsq | 0.908 | 0.885 | 0.909 | 0.913 | 0.891 | 0.913 |
| RMSE | 0.157 | 0.175 | 0.156 | 0.152 | 0.170 | 0.151 |

Notes: Dependent variable: the logarithm of wheat yield. Each column represents a separate regression model. Standard errors are displayed in parentheses and are clustered at the district-level. Stars indicate statistical significance: * *p* ≤ 0*.*1, ** *p* ≤ 0*.*05, ****p* ≤ 0*.*01.
All models include district fixed effects and state specific linear time trends. Columns 1, 3, 4 and 6 also include year fixed effects. Columns 3 and 6 include a quadratic precipitation term. Columns 1, 2 and 3 use a time-invariant long-run average irrigation share in each district and Columns 4, 5 and 6 use a time-varying lagged irrigation share over all previous years for each district-year pair.

Supplementary Table 5: Procurement and Production of Wheat in the main wheat-producing states

|  | Production  ( in 100,000 tons) | | Procurement by FCI and State Agencies ( in 100,000 tons) | | % of all India Procurement | Procurement as a percentage of total Production |
| --- | --- | --- | --- | --- | --- | --- |
|  | 2013-14 | 2014-15 | 2013-14 | 2014-15 |  |  |
| Bihar | 47.38 | 39.87 | 0 | 0 | 0 | 0 |
| Gujarat | 46.94 | 30.59 | 0 | 0 | 0 | 0 |
| Haryana | 118 | 103.54 | 58.73 | 65 | 23.29 | 55.8 |
| Madhya Pradesh | 129.37 | 171.04 | 63.55 | 70.94 | 25.33 | 44.8 |
| Maharashtra | 16.02 | 13.08 | 0 | 0 | 0 | 0 |
| Punjab | 176.2 | 150.5 | 108.97 | 116.41 | 42.45 | 69 |
| Rajasthan | 86.63 | 98.24 | 12.7 | 21.59 | 6.46 | 18.5 |
| Uttar Pradesh | 298.91 | 224.17 | 6.82 | 6.28 | 2.47 | 2.5 |

Source: Table 6 as provided in Reference (1). Original data is from the Ministry of Agriculture and the Food Corporation of India

Supplementary Table 6. Residual variation in weather

|  | GDD (10 days) | | EDD (10 days) | |
| --- | --- | --- | --- | --- |
|  | R-sq | SD of residual | R-sq | SD of residual |
| No FE |  | 20.19 |  | 3.132 |
| Year FE | 0.0927 | 19.23 | 0.168 | 2.857 |
| Dist FE | 0.701 | 11.04 | 0.676 | 1.784 |
| State Trend | 0.429 | 15.25 | 0.513 | 2.185 |
| Dist FE + Year FE | 0.794 | 9.166 | 0.844 | 1.239 |
| Dist FE + State Trend | 0.727 | 10.56 | 0.696 | 1.728 |
| Dist FE + Year FE+ State Trend | 0.817 | 8.644 | 0.854 | 1.198 |

Notes: Variation of GDD and EDD absorbed by fixed effects. Table summarizes regressions of GDD and EDD on various sets of fixed effects. Columns (a) report the R-square of the regression and Columns (b) report the standard deviation of the residuals or the remaining variation of GDD and EDD in the data

References

1. Chatterjee, S., & Kapur, D. Six puzzles in indian agriculture. In *India Policy Forum 2016* (Vol. 17, p. 13) (2017)
