# Supplementary material for: The role of irrigation in changing wheat yields and heat sensitivity in India
Source: Nat Commun. 2019 Sep 12;10:4144. doi: 10.1038/s41467-019-12183-9 (PMC6742628; doi:10.1038/s41467-019-12183-9)
Supplement: Supplementary file 2 — Reporting Summary [file 41467_2019_12183_MOESM2_ESM.pdf]

nature research

Corresponding author(s): Esha Zaveri

Last updated by author(s): Jul 26, 2019

Reporting Summary

Nature Research wishes to improve the reproducibility of the work that we publish. This form provides structure for consistency and transparency in reporting. For further information on Nature Research policies, see Authors & Referees and the Editorial Policy Checklist.

Statistics

For all statistical analyses, confirm that the following items are present in the figure legend, table legend, main text, or Methods section.

n/a	Confirmed	
☐	☒	The exact sample size (n) for each experimental group/condition, given as a discrete number and unit of measurement
☐	☒	A statement on whether measurements were taken from distinct samples or whether the same sample was measured repeatedly
☐	☒	The statistical test(s) used AND whether they are one- or two-sided<br>*Only common tests should be described solely by name; describe more complex techniques in the Methods section.*
☐	☒	A description of all covariates tested
☐	☒	A description of any assumptions or corrections, such as tests of normality and adjustment for multiple comparisons
☐	☒	A full description of the statistical parameters including central tendency (e.g. means) or other basic estimates (e.g. regression coefficient) AND variation (e.g. standard deviation) or associated estimates of uncertainty (e.g. confidence intervals)
☐	☒	For null hypothesis testing, the test statistic (e.g. F, t, r) with confidence intervals, effect sizes, degrees of freedom and P value noted<br>*Give P values as exact values whenever suitable.*
☒	☐	For Bayesian analysis, information on the choice of priors and Markov chain Monte Carlo settings
☒	☐	For hierarchical and complex designs, identification of the appropriate level for tests and full reporting of outcomes
☒	☐	Estimates of effect sizes (e.g. Cohen's d, Pearson's r), indicating how they were calculated

Our web collection on statistics for biologists contains articles on many of the points above.

Software and code

Policy information about availability of computer code

Data collection	*Provide a description of all commercial, open source and custom code used to collect the data in this study, specifying the version used OR state that no software was used.*
Data analysis	*Provide a description of all commercial, open source and custom code used to analyse the data in this study, specifying the version used OR state that no software was used.*

For manuscripts utilizing custom algorithms or software that are central to the research but not yet described in published literature, software must be made available to editors/reviewers. We strongly encourage code deposition in a community repository (e.g. GitHub). See the Nature Research guidelines for submitting code & software for further information.

Data

Policy information about availability of data

All manuscripts must include a data availability statement. This statement should provide the following information, where applicable:

- Accession codes, unique identifiers, or web links for publicly available datasets
- A list of figures that have associated raw data
- A description of any restrictions on data availability

The primary agricultural data used in this analysis are publicly available from the International Crop Research Institute for the Semi-Arid Tropics (ICRISAT) and their Village Dynamics in South Asia (VDSA) database (http://vdsa.icrisat.ac.in/vdsa-database.aspx). Observed temperature and precipitation data were acquired from the Indian Meteorological Department (http://www.imdpune.gov.in/ndc_new/Request.html). The data that support the findings of this study are available from the corresponding author upon request. The source data underlying Figs 1-3 are provided as a Source Data file

Field-specific reporting

Please select the one below that is the best fit for your research. If you are not sure, read the appropriate sections before making your selection.

☐ Life sciences ☐ Behavioural & social sciences ☒ Ecological, evolutionary & environmental sciences

For a reference copy of the document with all sections, see nature.com/documents/nr-reporting-summary-flat.pdf

Ecological, evolutionary & environmental sciences study design

All studies must disclose on these points even when the disclosure is negative.

Study description	the study assesses the impact of extreme temperature and irrigation on wheat yields using statistical analysis
Research sample	The primary agricultural data used in this analysis are publicly available from the International Crop Research Institute for the Semi-Arid Tropics (ICRISAT) and their Village Dynamics in South Asia (VDSA) database (http://vdsa.icrisat.ac.in/vdsa-database.aspx). Observed temperature and precipitation data were acquired from the Indian Meteorological Department (http://www.imdpune.gov.in/ndc_new/Request.html).
Sampling strategy	We include major wheat-producing states of Punjab, Haryana, Uttar Pradesh, Bihar, Rajasthan, Madhya Pradesh, Maharashtra and Gujarat in our analysis. Together, these states account for over 90 % of national wheat production. We use data from 1970-2009 in our analysis since a majority of observations for the major wheat-producing states are available in this time-period.
Data collection	The historical agricultural data used in our analysis was acquired from the International Crop Research Institute for the Semi-Arid Tropics (ICRISAT) and their Village Dynamics in South Asia (VDSA) database, which collates data from State Directories of Agriculture, State Bureaus of Economics and Statistics, State Planning Departments, various Agricultural Censuses, and government reports. Observed temperature and precipitation data were acquired from the Indian Meteorological Department at a spatial resolution of 1° x 1° (temperature) and 0.25° x 0.25° (precipitation).
Timing and spatial scale	We use data from 1970-2009 in our analysis since a majority of agriculture-related observations for the major wheat-producing states in the ICRISAT database are available in this time-period
Data exclusions	We include major wheat-producing states of Punjab, Haryana, Uttar Pradesh, Bihar, Rajasthan, Madhya Pradesh, Maharashtra and Gujarat in our analysis. Together, these states account for over 90 % of national wheat production.
Reproducibility	*Describe the measures taken to verify the reproducibility of experimental findings. For each experiment, note whether any attempts to repeat the experiment failed OR state that all attempts to repeat the experiment were successful.*
Randomization	*Describe how samples/organisms/participants were allocated into groups. If allocation was not random, describe how covariates were controlled. If this is not relevant to your study, explain why.*
Blinding	*Describe the extent of blinding used during data acquisition and analysis. If blinding was not possible, describe why OR explain why blinding was not relevant to your study.*

Did the study involve field work? ☐ Yes ☒ No

Reporting for specific materials, systems and methods

We require information from authors about some types of materials, experimental systems and methods used in many studies. Here, indicate whether each material, system or method listed is relevant to your study. If you are not sure if a list item applies to your research, read the appropriate section before selecting a response.

Materials & experimental systems

n/a	Involved in the study
☒	☐ Antibodies
☒	☐ Eukaryotic cell lines
☒	☐ Palaeontology
☒	☐ Animals and other organisms
☒	☐ Human research participants
☒	☐ Clinical data

Methods

n/a	Involved in the study
☒	☐ ChIP-seq
☒	☐ Flow cytometry
☒	☐ MRI-based neuroimaging
